# Psychological adjustment and quality of life in children and adolescents following open-heart surgery for congenital heart disease: a systematic review

**DOI:** 10.1186/1471-2431-9-6

**Published:** 2009-01-22

**Authors:** Beatrice Latal, Susanne Helfricht, Joachim E Fischer, Urs Bauersfeld, Markus A Landolt

**Affiliations:** 1 University Children's Hospital Zurich, Child Development Centre, Steinwiesstrasse 75, 8032 Zurich, Switzerland; 2 Heidelberg University, Mannheim Medical Faculty, Institute of Public Health, Social and Preventive Medicine, 68072 Mannheim, Germany; 3 University Children's Hospital Zurich, Department of Pediatric Cardiology, Steinwiesstrasse 75, 8032 Zurich, Switzerland; 4 University Children's Hospital Zurich, Department of Psychosomatics and Psychiatry, Steinwiesstrasse 75, 8032 Zurich, Switzerland

## Abstract

**Background:**

Children with congenital heart defects (CHD) requiring open-heart surgery are a group at high risk for health-related sequelae. Little consensus exists regarding their long-term psychological adjustment (PA) and health-related quality of life (QoL). Thus, we conducted a systematic review to determine the current knowledge on long-term outcome in this population.

**Methods:**

We included randomized controlled trials, case control, or cohort studies published between 1990–2008 evaluating self- and proxy-reported PA and QoL in patients aged between two and 17 years with a follow-up of at least two years after open heart surgery for CHD.

**Results:**

Twenty-three studies assessing psychological parameters and 12 studies assessing QoL were included. Methodological quality of the studies varied greatly with most studies showing a moderate quality. Results were as follows: (a) A considerable proportion of children experienced psychological maladjustment according to their parents; (b) studies on self-reported PA indicate a good outcome; (c) the studies on QoL suggest an impaired QoL for some children in particular for those with more severe cardiac disease; (d) parental reports of psychological maladjustment were related to severity of CHD and developmental delay.

**Conclusion:**

A significant proportion of survivors of open-heart surgery for CHD are at risk for psychological maladjustment and impaired QoL. Future research needs to focus on self-reports, QoL data and adolescents.

## Background

Congenital heart disease (CHD) occurs in 4–12 per 1,000 live births [1,2]. More than one third of the affected children are born with "critical heart disease" denoting malformations, which acutely threaten life and necessitate palliative or corrective surgery in early life [3]. Advances in surgical and postoperative support techniques have significantly reduced the mortality and have lead to acceptable mid- and long-term cardiac outcome even for children with complex CHD, premature or low-birth-weight children [4-8]. Various risk factors for long-term morbidity have been identified. Particularly, cardiopulmonary bypass surgery is associated with inflammatory responses, renal, myocardial, and neurological injury [9] and may consequently lead to neurodevelopmental impairments [10,11]. While open-heart surgery can correct or palliate CHD, it also represents the challenge of psychologically adjusting to the surgical event and associated side effects for the child and the parents. Moreover, the question arises as to how quality of life in these patients is affected by the surgical event.

There is an increasing body of literature addressing these questions. Many studies have examined neuropsychological functioning, behavior and quality of life in children and parents. However, methodological shortcomings make it difficult to compare studies. They vary greatly in study design, inclusion criteria, assessment of risk factors, duration of follow-up, attrition and in particular in outcome measures. Thus, a systematic review of the existing literature is needed to summarize the evidence on psychological outcome and quality of life and the pertinent risk factors.

To facilitate the understanding of this review, the relevant terminology is briefly introduced: The assessment of *psychological adjustment *incorporates a range of outcome measures including behavioral, emotional or psychosocial constructs. A common approach differentiates between internalizing and externalizing behavior difficulties [12]. The former are characterised by symptoms of anxiety, depression, and withdrawal, while the latter include delinquent, aggressive, and show off behavior.

*Quality of life (QoL) *is a multidimensional construct integrating an individual's subjective perceptions of physical, social, emotional and cognitive functioning [13]. In the context of patient populations this is referred to as health-related quality of life. Traditionally, QoL in cardiac patients has been estimated by objective indices related to health outcomes such as cardiopulmonary exercise capacity [14], exercise tolerance [15], or the New York Heart Association classification. However, meanwhile there is a general agreement that these indices alone do not suffice to reflect QoL in cardiac patients in all its facets [16].

Psychological adjustment and QoL can be assessed by means of self- or proxy-reports. Generic as well as disease-specific assessment instruments exist. The former are used with any patient population independent of their disease, while the latter assess disease-specific issues. Some instruments combine generic with disease-specific items.

This review aims to systematically assess studies on psychological adjustment and QoL in children and adolescents with CHD undergoing cardiopulmonary bypass surgery. Also, associations between medical, individual and familial risk factors and the long-term outcome were evaluated.

## Methods

### Search strategy and selection criteria

**Figure 1:**
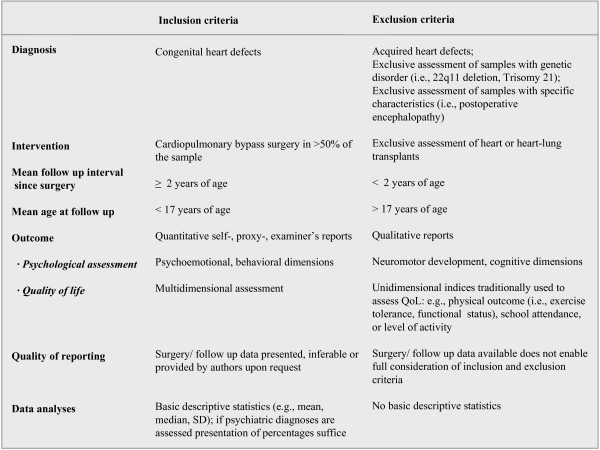
**Overview of inclusion and exclusion criteria**.

For the period between January 1990 and July 2008 a literature search was conducted via EMBASE, MEDLINE, CINAHL, and PsycInfo to identify eligible studies and review articles. We chose this rather short period to reduce the variance in observed outcomes due to time-dependent advances in surgical techniques. Additionally, databases of dissertations were searched for (ProQest, NDLTD). Great effort was spent to identify the best search strategy for the respective databases aiming at a good ratio of overall hits and eligible studies. An initial search used the identifiers congenital heart disease, congenital heart defect*, the main diagnostic categories of CHD known to require cardiopulmonary bypass surgery (e.g., tetralogy of Fallot, transposition of the great arteries, hypoplastic- left heart syndrome, etc.), p*ediatrics, child*, adolescent*, adolescence, open-heart surgery, cardiac surgery, cardiopulmonary bypass surgery, heart-lung machine, arterial switch, atrial switch, Mustard, Senning, Norwood, Fontan, circulatory arrest, hypothermia, low flow, full flow, behavio*r, psychological, psychosocial, psychiatric, psychopathology, mental health, health, quality of life, health-related quality of life, adjustment, adaptation, emotional, development*, neurodevelopment*. The Boolean operator "*and*" was used to combine identifiers for patient population, intervention, and outcome. The operator "*or*" was used to combine identifiers within those search areas. Adjustments in search strategies were made for CINAHL and PsycInfo as the initial search strategy was too specific and resulted in few hits only. Overall, the electronic search resulted in 732 hits. Reference lists of relevant studies and reviews were examined to identify other pertinent articles. Furthermore, investigators from the field were contacted to enquire about unpublished data. One reviewer (SH) pre-selected 84 articles according to the information obtained from titles or abstracts. Two independent reviewers (BL, MAL) blinded to the origin of the articles checked the full texts of these articles for inclusion according to a standardized predefined checklist. Inclusion and exclusion criteria are presented in Figure 1. Since there were few studies where all patients were operated with open-heart surgery, we included those in which the majority of patients (> 50%) were operated with open-heart surgery. The follow-up period of at least 2 years was chosen to exclude the effect of acute psychological distress. In addition, we only focused on a pediatric population and thus included studies in which patients were on average younger than 17 years at the time of assessment. The other inclusion criteria were created to enable a meaningful conclusion regarding main effects and risk factors for PA and QoL. In case of missing information, corresponding authors were contacted. Accordingly, 23 studies on psychological adjustment and 12 studies assessing QoL were included. We did not identify any unpublished data eligible for inclusion.

### Quality assessment

**Figure 2:**
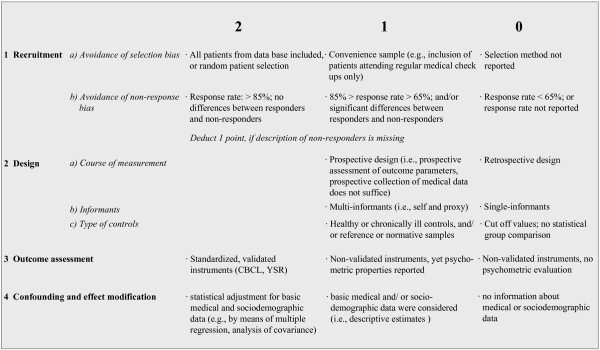
**Overview of methodological quality rating criteria for included studies**.

Methodological quality of the included studies was rated by the two blinded reviewers (BL, MAL). A standardized checklist with criteria relating to recruitment, study design, operationalisation of outcome, and statistical analyses was used. Each criterion was rated on a two- or three-step scale indicating the degree of fulfillment with the respective criterion. The checklist is presented in Figure 2. Overall scores ranged from 0 to 11 points with higher scores indicating a better study quality. Interrater reliability was excellent (Cohen's kappa = 0.91). In case of disagreement between the two reviewers, consensus was achieved. In addition, three quality groups were formed: good (score 9–11), moderate (score 6–8) and poor (score 0–5) quality.

### Data extraction and synthesis

Two independent reviewers (BL, MAL) extracted data on relevant outcomes, characteristics of study design, sample, and surgical technique. Study samples differed significantly with regard to severity of heart defect, age at time of surgery, number of operations, postoperative course, or age at follow up. Moreover, as summarized in Table 1, the operationalization of psychological outcome and QoL varied greatly as different constructs such as behavior, self esteem or mental health had been evaluated. Heterogeneity of assessment methods and differing presentation of the results did not permit formal meta-analysis. Therefore, findings are summarized in a narrative way. An overview of study characteristics, methodological quality ratings, and study results is presented in Additional file 1 and 2 [see Additional file 1 and 2]. The mean (standard deviations; range) quality rating scores for studies on PA was 7.04 (1.33; 4–10); for studies on QoL it was 6.54 (1.45; 5–9). Overall, most studies reporting on psychological adjustment and QoL were of moderate quality (78%; 55% respectively); two studies in each area of research were rated as high quality studies (9%; 18% respectively) [see Additional file 1 and 2].

**Table 1:** Overview of questionnaires and interviews applied in the reviewed studies to assess psychological adjustment and quality of life

**Diagnostic instruments**	**Studies **(Reference numbers)
**Psychological adjustment – semi-structured interviews**	
Child Assessment Schedule (CAS)	[19]
Child Behavior Problems Interview (CBPI)	[18]
**Psychological adjustment – proxy-report measures**	
Child Behavior Checklist (CBCL)	[19-22,24-33,35,36,38,39]
Teacher's Report Form (TRF)	[21,22,33]
Vineland Adaptive Behavior Scales (VABS)	[29,40]
**Psychological adjustment – self-report measures**	
Child Depression Inventory (CDI)	[23,36]
Children's Manifest Anxiety Scale-revised (CMAS-R)	[23]
Fear Survey Schedule for Children-revised (FSSC-R)	[23]
Frankfurt Scales of Self Concept (FSKS, German)	[34]
High School Personality Questionnaire (HSPQ)	[34]
I Think I Am Scale (ITIAS)	[18]
Self Perception Profile for Children	[36]
State-Trait Anxiety Inventory (STAI)	[34,36]
Youth Self Report (YSR)	[27,34,35]
**Quality of life**	
Child Health Questionnaire, parent form (CHQ-PF)	[41,43,46]
Child Health Questionnaire, child form (CHQ-CF)	[44]
Inventory for the Assessment of the Quality of life in Children and Adolescents (IQLC)	[24]
Lindström Model of Quality of Life (LMQL)	[42]
LQ-KID and LQ-KID-E	[49]
Pediatric Quality of life Inventory (PedsQL)	[28,47]
Pediatric Cardiac Quality of Life Inventory (PCQLI)	[50]
TNO-AZL Child Quality of Life Questionnaire (TACQOL)	[45,48]

## Results

### Psychological adjustment

#### Incidence of clinically significant maladjustment

Two studies examined psychiatric diagnoses according to the DSM-IV [17] by means of respondent-based semi-structured interviews: Rates of psychiatric disorders for children with surgically corrected transposition of the great arteries [18] and children with severe cyanotic defects after surgery [19] amounted to 19% and 46%, respectively. However, in children with transposition of the great arteries [18], the corrective surgery was performed with the Senning or Mustard procedure, which does not achieve a complete repair of the underlying heart defect.

Almost all studies examined behavior according to parental reports assessed by the Child Behavior Checklist [12]. Reported rates of maladjustment ranged from 5% to 41%. Except for one study in which children with cyanotic-corrected heart disease (transposition of the great arteries, tetralogy of Fallot) had low maladjustment rates [20], all rates were significantly higher than those reported for either a normative group or another type of control group (i.e., siblings or healthy controls).

Two studies published results on teacher reports of child maladjustment. Compared to parents, deviant behavior was less frequently reported by teachers, with rates ranging from 4% [21] to 14% [22]. These rates were not significantly different from controls.

#### Proxy-reported psychological functioning

Besides assessing the incidence of clinically significant maladjustment another approach is to assess mean levels of postoperative psychological functioning across samples. The majority of studies reported on proxy-reported psychological functioning in children with CHD. *Parent-reported *overall behavioral functioning was consistently more affected than that of normative samples or control groups. Psychological difficulties consisted predominantly of internalizing symptoms [21,23-27] Three studies also observed significantly more externalizing symptoms; one study for children with surgically corrected transposition of the great arteries compared to norms [24]; one for children with hypoplastic left heart syndrome compared to children with transposition of the great arteries [28] and the third for children with ventricular septal defect compared to children with atrial septal defect [27]. Except for one [28] all of the above studies were of moderate methodological quality.

In contrast, four studies did not detect any significant differences in proxy-reported psychological functioning [22,29-31]. Of these, one study had received a high-quality methodological rating [31]. Interestingly, one study with a moderate quality rating reported a better behavioral outcome in children with d-transposition of the great arteries after *arterial *switch operation [32] compared to controls. However, these children were only two years of age at the time of assessment.

*Proxy-reports *by teachers did not reveal any significant differences in behavior between children with operated heart defects and controls [21,22] except for one study, which reported more behavioral difficulties in operated children compared to controls [33].

#### Self-reported psychological functioning

Seven studies evaluated self-reported psychological functioning. While three studies did not observe significant differences with regard to overall fear and anxiety [23], self-esteem [18] or overall behavioral difficulties [34], one study found significantly more behavioral difficulties in a sample consisting predominantly of adolescents [35]. Another study reported a higher rate of self-reported depressive symptoms in school-age children [36]. Interestingly, in a Dutch study, young adolescents with CHD reported less rule- breaking behavior than healthy controls [27]. Except for one study with a low methodological rating [34], all other studies were of moderate or good methodological quality.

#### Risk factors for psychological malfunctioning

The *type of heart defect *was unrelated to *proxy-reported *psychological functioning in most studies [26,29,35,37-39]. Accordingly, neither the presence of cyanosis [20,23], nor cardiac function [18] was associated with self-reported psychological adjustment. In contrast, two studies found an effect of the type of heart defect on behavior. One could show that parents of children with ventricular septal defect reported more behavioral problems than parents of children with atrioseptal defect or pulmonary stenosis [27]. Another study demonstrated that only children with a hypoplastic left heart syndrome had psychological maladjustment compared to those with a transposition of the great arteries [28]. In line with these findings is that *parents *of children with cyanosis [23], or with reduced physical capacity [19], reported psychological maladjustment following surgery in their child. Correspondingly, children in need for further surgical intervention were more likely to suffer from co-morbid psychiatric illness than those without this prospect according to *parental reports *[18].

*Surgery-related *deep hypothermic circulatory arrest was associated with poorer proxy-reported psychological outcome compared to low or full flow bypass procedures [32,39]. Additionally, the duration of deep hypothermic circulatory arrest correlated with the degree of proxy-reported psychological difficulties in one study [24], yet not in another study [40]. Peri- and postoperative cardiovascular insufficiency related to poorer proxy-reported behavioral outcome [24]. Older age at surgery and a higher number of surgeries were related to reduced proxy-reported psychological functioning in one study [39]. Another study did not find support for such a relationship [40].

*Individual characteristics *such as postoperative developmental delay were consistently related to child psychological maladjustment in *parental reports *[24,32,35,37]. Yet, self-reported psychological adjustment was not associated with measures of intelligence [18,35]. Sex differences in overall psychological adjustment were not observed [37,39].

*Family characteristics* early parental distress was related to *proxy-reported *behavioral difficulties in children with operated transposition of the great arteries [31] and maternal anxiety was related to self-reported fear in the child as well as to *proxy-reported *behavioral maladjustment [23]. In addition, poor parental control skills, single parent status, high maternal worry about the child and increased levels of maternal psychological symptoms were all related to poorer behavioral adjustment in children with a variety of CHD [20]. Interestingly, one study demonstrated that early social support for the family was unrelated to long-term psychological adjustment in the operated child [31].

### Quality of life

Additional file 2 [see Additional file 2] gives an overview of the results of the 12 studies reporting postoperative QoL. In four of these studies QoL in children with operated CHD was comparable to that in normative samples according to proxy- [28,41], self- [24] or combined reports [42]. These studies received quality ratings between 5 and 9 points. Another study observed a normal *proxy-*reported QoL in more than 90% of their sample based on cut-off values, yet this result was not based on inferential statistics [43]. One study observed a better self-reported QoL in a large sample of children with transposition of the great arteries undergoing surgery compared to healthy norms [44]. In this sample, corrective surgery included the atrial as well as the arterial switch operation. Children with arterial switch operation showed a better functioning compared to those with atrial switch. In contrast, four studies reported an impaired QoL in many self- and most *proxy*-reported dimensions [45-48]. Methodological quality in these studies varied between 5 and 9 points. Two studies did not report overall QoL, but examined the agreement between parental and self rated QoL [49] and the influence of two ventricle versus single ventricle physiology [50].

Two studies compared QoL of children with CHD with that of children with other chronic illnesses. In one study children with CHD after surgery experienced a better *proxy-reported *QoL [47], yet the opposite was found in another study [41].

As QoL is a multidimensional construct various subdimensions were evaluated across studies. A direct comparison between studies, however, cannot be made as different measures and concepts of QoL have been used (see Table 1).

#### Risk factors for impaired quality of life

*The type of heart defect *did not relate to QoL in a heterogeneous diagnostic sample [48] and in a small sample of children with transposition of the great arteries or hypoplastic left heart syndrome [28]. However, two studies found a lower QoL in children with more complex malformations [44,47]. In a sample of children with single ventricle anatomy only few cardiac-specific factors were found to be related to QoL [46].

Considering *surgery related factors *cardiopulmonary bypass duration was inversely related to QoL in three studies [24,44,45] but not in other studies [43,46]. Also, conflicting data exist on the effects of duration of circulatory arrest [41,43,44] or the number of cardiac surgeries [41,46,47]. No effects on QoL were found for vital organ support technique [41], cooling temperature [44], or age at surgery [43,46]. Postoperative complications, length of hospital stay and current need for cardiac medication were negatively related to QoL in two studies [45,46]. 

*Individual characteristics* sociodemographic factors such as sex [41,48] or socioeconomic status [41,45,47] were not associated with QoL in children. However, Landolt et al. [45] found a higher self-reported QoL in boys compared to girls. Older age at follow up assessment was associated with a better QoL in two studies [47,48]. Also, a higher IQ was related to better psychosocial QoL in one study [41]. 

*Family characteristics* children of parents who remained unemployed due to the child's health condition or children of families with a low income experienced a lower QoL than controls [46]. Finally, adverse family relationship [45] and parental stress at follow up [43] were both found to be negatively related to the psychosocial dimensions of QoL.

## Discussion

This review is the first to systematically summarize the literature of the last 18 years on psychological adjustment and quality of life of children and adolescents with CHD following cardiopulmonary bypass surgery. Moreover, potential risk factors for psychological maladjustment and impaired quality of life are reported.

### Proxy-reported long-term outcome

Studies assessing the incidence of psychiatric diagnoses, although few, as well as studies investigating the rates of clinically significant behavioral symptoms have demonstrated that a considerable proportion of these children experience psychological maladjustment according to their parents. The observed proportion of children with a psychiatric diagnosis [18,19] is comparable to children with other chronic illnesses, who have a two-fold higher risk than children in the general population [51,52]. Likewise, significantly more children with operated defects displayed behavior outside a normative range [21,22,24,30,35]. In the same line, studies assessing mean levels of psychological functioning found significantly more psychological difficulties in operated children compared to controls [21,23,24,33,35,37,40]. As an exception, parents of children with surgically corrected transposition of the great arteries consistently reported good or even better psychological adjustment [31,32,41] than controls. This good outcome may be due to the excellent prospect of correcting this malformation by means of arterial switch operation. A lower rate of genetic disorders in this subgroup of patients may also contribute to this favorable outcome. As the latter findings originated from the same institution and are partly based on overlapping samples, they need to be confirmed by other groups.

Interestingly, teachers judged the behavior and functioning of children with CHD similar to that of control children [21,22]. Although these findings need replication, they point out the discrepancy between parental reports and teacher reports. The results indicate that the children's difficulties are less apparent within the schooling context. This may be due to the fact that children are reported to show predominantly internalizing problems, which are less likely to be detected by teachers than by parents. Alternatively, this result may highlight the importance of the negative influence of parental anxieties with regard to their subjective perception of their children.

### Self-reported long-term outcome

To date, there are only few studies that assessed self-reported psychological adjustment in children after cardiopulmonary bypass surgery. Thus, available results do not allow for a final conclusion but provide further directions of research. Three studies did not find differences in self-reports of children with operated heart defects compared to a reference group, thus contrasting proxy reports [18,23,34]. The divergence between parents and their children (and teachers), the so-called cross-informant variance, has frequently been reported across different clinical samples [53]. It may reflect differing, yet equally important, realities [54] and highlights the need for outcome assessments using multiple informants. Only two studies observed a significant degree of self-reported behavioral difficulties [35,36]. Since the study by Utens et al. comprised a large proportion of adolescent cases, this finding does not contradict other results that are based on self-reports in children. Yet, it may indicate a decline in psychological adjustment associated with the onset of puberty and the increasing academic demands. Clearly, more research is needed to confirm these findings.

### Quality of life

Current data suggest that children with CHD undergoing cardiopulmonary bypass surgery may be faced with an impaired QoL at follow-up [45-48]. Impairments are more frequently reported by parents than by the patients themselves. Notably, in 4 of the 12 reviewed studies, a normal QoL was found. Children with operated transposition of the great arteries may be overrepresented in the reviewed studies as most samples comprised children with this defect only [24,41,44] or included a subsample of these children [43,48]. Thus, findings may not be applicable to the remaining population of children with CHD.

### Risk factors for long-term outcome

To date, there is insufficient and conflicting data to attribute long-term outcome to the various risk factors assessed. As only few studies evaluated the same risk factors, findings are preliminary and demand replication. Moreover, the evaluation of risk factors often did not adhere to strict statistical standards, i.e., by adjusting for confounding variables by means of multivariate analysis.

The cardiac diagnosis does not seem to be a particular risk factor for psychological maladjustment after cardiopulmonary bypass surgery. This has been demonstrated in numerous studies across different ages [26,29,35,37-39,41,45]. Nevertheless, physical correlates of the heart defects (such as cardiac insufficiency mandating cardiac medications or arrhythmias during follow-up) or other physical problems such as pulmonary problems may lead to reduced *proxy-reported *psychological adjustment and QoL [19,23,45-47]. Such a relationship was not detected for child self-reports [18,23].

While surgery itself may pose a risk for long-term outcome, current research has attempted to assess the effects of various organ support techniques, duration of surgery [24,32,39-41,44,45] and length of hospital stay [45]. However, potential confounders such as depth of hypothermia or postoperative complications must be carefully controlled for before a conclusion can be derived. Likewise more data are needed addressing the conflicting current findings.

Developmental delay is a child-specific risk factor that has consistently been found to relate to *proxy-reported *psychological maladjustment following heart surgery [24,32,35,37,41]. Thus, parents of children with language difficulties or cognitive delay reported more psychological difficulties in their children. In contrast, this association was not found for self-reported psychological adjustment. Other risk factors such as sex, education or socioeconomic status have rarely been investigated.

Finally, the parents of children with operated heart defects themselves may play an important role with regard to the child's long-term psychological adjustment [23,31] and quality of life [43,45,46]. However, more research is needed to further evaluate the role of parental adjustment for the long-term outcome and QoL in their children. Also, the causal rather than the correlational nature of this relationship warrants empirical support.

### Limitations and implications for future research

With regard to the methodology of the reviewed studies, several limitations were identified. Only four out of 35 studies were found to be of high methodological quality according to a self-developed rating system. All but one study [31] applied a cross-sectional or retrospective design. This approach does not allow to describe the dynamic course of psychological adjustment or QoL in relation to surgery. Neither does the use of normative data or healthy control groups suffice to causally attribute group differences to the surgical procedure itself; they may be associated with the chronic disease itself [41,47]. Thus, a control group design should account for *a) *the effect of a high-risk surgical intervention, and *b) *the effect of living with CHD. Even if these standards are met, results still need to be discussed in the light of potential confounding, i.e., less severe illness in controls.

In this systematic review, we did not include studies that only focused on children with chromosomal anomalies. However, we did not find a study that only addressed this subgroup. The reviewed studies typically excluded patients with chromosomal anomalies and non-cardiac comorbidities. Because cardiac defects frequently result from chromosomal disorders and because neurological disability is one of the major risk factors associated with cardiopulmonary bypass surgery, the exclusion of such patients may have biased findings towards better outcome. Thus, less restrictive exclusion criteria and the systematic use of subgroup analysis will allow to determine QoL and psychological adjustment in these children.

Other limitations relate to the operationalization of outcome variables. As most studies assessed psychological adjustment by means of the Child Behavior Checklist [12] well-known shortcomings of the scale within the context of chronic disease must be addressed [55]. While this instrument has been validated for a psychiatric population, its suitability for chronically ill children is unclear. The optimal assessment instrument should allow for a clear distinction between symptoms primarily associated with the disease and those associated with consequences of the disease validated for the target population. To the best of our knowledge, no such scale exists for the assessment of psychological adjustment in children with chronic diseases. In contrast, such progress has been made with regard to the psychometric assessment of quality of life. Recently, the PedsQL [56] has been supplemented with a cardiac module [57], and is currently under validation. In addition, a large US research group has published the Pediatric Quality of Life Inventory (PCQLI), which is a disease specific QoL measure for children with CHD [50]. The PCQLI has patient and parent-proxy forms, covers a wide age range and showed very promising data in the multi-center validation study. Certainly, future studies on QoL in children with CHD should incorporate these new disease specific measures.

## Conclusion

This review demonstrated that a significant proportion of children with CHD experience psychological maladjustment following cardiopulmonary bypass surgery. Children with more severe heart defects, or those in need of future surgical interventions and children with neurodevelopmental impairment are at particular risk for maladjustment. The QoL of these children appears also to be affected, particular with regard to parental reports. However, the literature on this important outcome is still emerging and disease-specific instruments have just been published. Importantly, parental well-being seems to be related to psychological adjustment in these children. This calls for an integrated approach to family support, taking the child's individual needs into account as well as the needs of the parents.

## Competing interests

The authors declare that they have no competing interests.

## Authors' contributions

BL and MAL participated in the selection and review of studies and the writing of the manuscript. SH participated in the selection and review of studies and drafted the manuscript. JEF and UB participated in the interpretation of findings and critically reviewed the manuscript. All authors read and approved the final manuscript.

## Pre-publication history

The pre-publication history for this paper can be accessed here:



## Supplementary Material

Additional File 1 **Table presenting psychological adjustment in children and adolescents with CHD after CPB surgery grouped by major outcome variables and methodological quality.** The table gives an overview of studies assessing psychological adjustment in children with congenital heart defects after bypass surgery, studies are grouped by major outcome variables and methodological quality. Click here for file

Additional File 2 **Table presenting quality of life in children and adolescents with CHD after CPB surgery grouped by major outcome variables and methodological quality.** The table gives an overview of studies assessing quality of life in children with congenital heart defects after bypass surgery, studies are grouped by major outcome variables and methodological quality. Click here for file
